# A Hard Nut to Crack: Reducing Chemical Migration in Food-Contact Materials

**DOI:** 10.1289/ehp.123-A174

**Published:** 2015-07-01

**Authors:** Nate Seltenrich

**Affiliations:** Nate Seltenrich covers science and the environment from Petaluma, CA. His work has appeared in *High Country News*, *Sierra*, *Yale Environment 360*, *Earth Island Journal*, and other regional and national publications.

When we buy food, we’re often buying packaging, too. From cherries to Cheez-It® crackers, modern foods are processed, transported, stored, and sold in specialized materials that account, on average, for half the cost of the item, according to Joseph Hotchkiss, a professor in Michigan State University’s School of Packaging. Consumer-level food packaging serves a wide range of functions, such as providing product information, preventing spoilage, and protecting food during the journey from production to retail to pantry, fridge, or freezer. That’s why food producers lavish so much time and money on it.

But what happens when these valuable and painstakingly engineered containers leach chemicals and other compounds into the food and drink they’re designed to protect? Such contamination is nearly ubiquitous; it happens every day, everywhere packaged food is found, with all common types of packaging, including glass, metal, paper, and plastic.^1^^,^^2^^,^^3^^,^^4^

**Figure:**
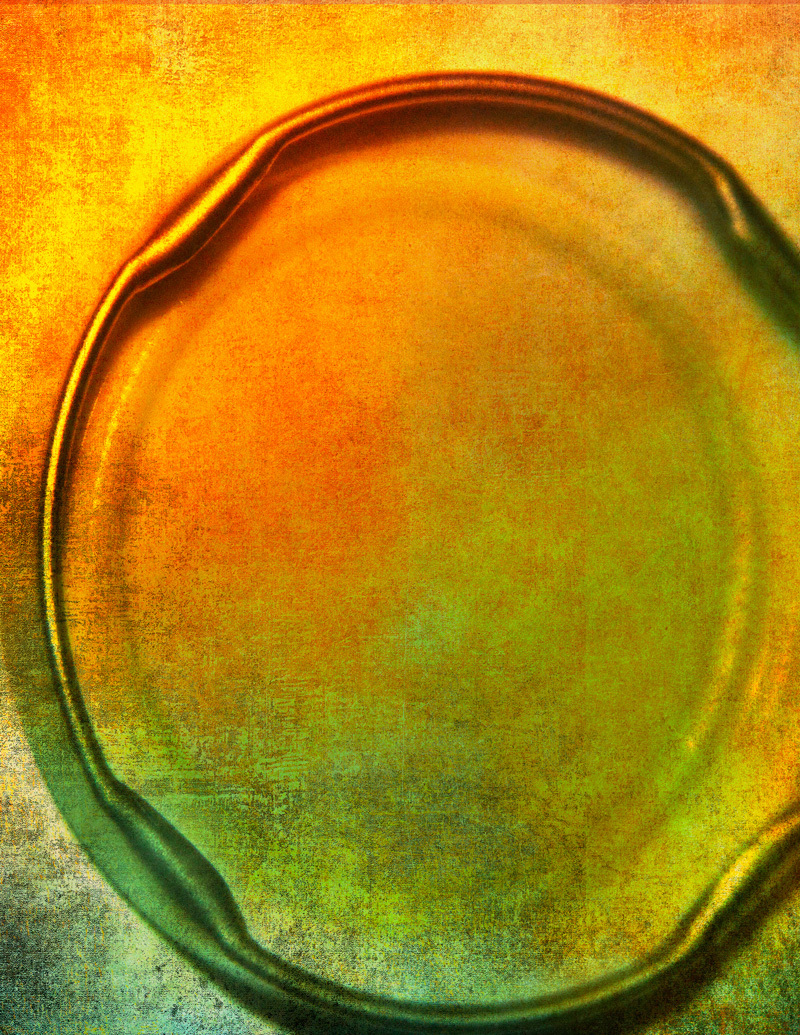
Many manufacturers are eager to alleviate the problem of chemical migration from food packaging, but progress in identifying viable alternative materials has been incremental at best. © Joseph Tart/Brogan & Partners

Even as awareness of the issue grows, large-scale solutions that are scientifically and financially viable remain out of reach. The challenges in reaching them are many. Yet some of the world’s leading health authorities and largest food producers are working toward fixes (and in cases already deploying them), despite the absence of scientific consensus or regulatory requirements around most food-packaging chemicals of concern.

## The Winding Path of Chemical Replacement

Due primarily to consumer demand, health concerns represent the largest force driving innovation within the food-packaging industry today, Hotchkiss says. “I believe the safety issues will continue to grow, and those who can assure consumers that they are concerned about it and are doing what they can to address it will be rewarded in the marketplace,” he says. “Those that don’t will be punished in the marketplace.”

People around the world are familiar with bisphenol A (BPA) and concerns about its migration into food and drink from plastic bottles, metal cans, and other consumer products. To date U.S. and European authorities have concluded, based on the available evidence, that the levels of BPA that currently occur in foods are safe for all consumers.^5^^,^^6^ Other scientists suggest the experimental evidence for BPA’s adverse health effects is strong enough to warrant removing the chemical from food-use applications as a precaution.^7^^,^^8^ In recent years U.S. manufacturers voluntarily abandoned the use of BPA in baby bottles, sippy cups, and infant-formula packaging, and the U.S. Food and Drug Administration (FDA) formally ended its authorizations of these uses thereafter.^9^

Beyond our borders, several other countries have banned BPA from some infant products, including Canada, the European Union, South Africa, China, Malaysia, Argentina, Brazil, and Ecuador.^10^ France went even further with its recently implemented ban of BPA from all packaging, containers, and utensils that come into contact with food.^11^

The BPA debate illuminates many of the challenges involved in stemming chemical migration. As France recognized with its ban, prohibitions for baby products alone don’t address the fact that BPA exists in countless consumer products and food-packaging materials to which infants and expectant mothers,^12^ among other susceptible populations, may still be exposed—such as metal beverage and food cans, which are often lined with BPA-based epoxy resins.^13^

BPA is just one of many known or suspected endocrine disruptors commonly found in food packaging that can migrate into food and drink.^14^^,^^15^ Furthermore, endocrine disruptors from plastics are far from the only class of potentially harmful chemicals that can leach into food or drink from food packaging; depending on factors including temperature, storage time, and physicochemical properties, a wide variety of compounds—including components of coatings and films, adhesives and glues, and inks and pigments—can migrate from packaging materials.^16^^,^^17^

For these reasons, Laura Vandenberg, an assistant professor of environmental health at the University of Massachusetts Amherst, believes most existing bans on BPA do little to ensure food safety. “This was a very empty victory, I think, to focus on BPA and baby bottles,” she says.

## Alternative Plastics

Sure enough, in some applications BPA was replaced with other bisphenols, including BPS and BPF, which laboratory experiments indicate have estrogenic effects at least as pronounced as those of BPA.^18^ In others, including baby bottles, polycarbonates were replaced by alternative plastics with migration issues of their own.^19^

Chemists are now on the hunt for effective alternatives to BPA. To date no one has identified any drop-in fixes that will work in all the same applications, for the same or a lesser cost, with an established lack of estrogenic activity (now known in the marketplace as “EA-free”). But partial solutions are beginning to appear.

One of the most widely available is a polymer called Tritan that can replace traditional polycarbonate in clear, hard plastics used for water and baby bottles. According to its manufacturer, Eastman Chemical Company, Tritan is free of estrogenic activity within the human body.^20^

Not everyone agrees. In 2011 a pair of affiliated firms called PlastiPure and CertiChem published a study showing the potential for endocrine disruption in Tritan.^21^ This sparked a lawsuit from Eastman, which it later won.^22^ At the core of the case was the question of how best to detect and define estrogenic activity; the two sides used different tests that each insisted was accurate.^23^

Tritan is still used widely in hard-plastic bottles sold by Nalgene, CamelBak, Nathan, and other brands, while PlastiPure and CertiChem continue to support the development of other alternative plastics and products, including food packaging, says chief economic officer Mike Usey. In addition to testing and consulting, the sister companies will soon expand into product development, Usey says. “We’ve had so much interest in the last year and a half from consumers for safer products, and a lack of traction with manufacturers, that we’ve decided to spin off a product company.”

But full-scale solutions remain at least a few iterations away, says John Warner of the Warner Babcock Institute for Green Chemistry. “Something like reinventing plastic isn’t going to happen in a day, a month, or a year,” he says. “This isn’t a matchmaking game. It’s not like the solutions are out there, if only the companies could be matched up with those solutions. I really feel we are inventions away from success.”

Much of Warner’s personal research centers on developing biobased plastics (i.e., derived from renewable biomass sources) that are safer, cheaper, and as effective as traditional fossil-fuel plastics for food packaging. However, plant-based plastics still may contain some of the same harmful additives and manufacturing by-products (known as non-intentionally added substances) that can migrate into food and drink.

These plastics do offer one distinct advantage, Warner says: “Because bioplastics are new, they have less of an incumbent history, so designers, inventors, and developers can create a better formulation of additives that have less impact on human health and the environment.” In other words, although it doesn’t guarantee success, there may be more opportunity for creativity and innovation around bioplastics than with traditional plastics that are more entrenched in industry, he speculates.

**Figure:**
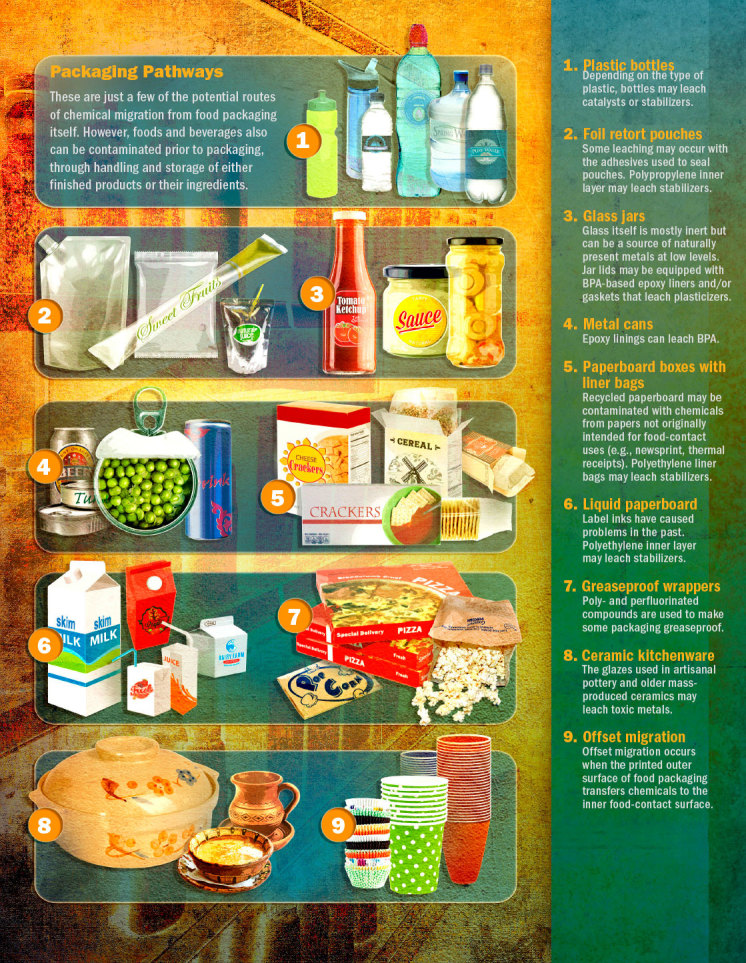
**Packaging Pathways** These are just a few of the potential routes of chemical migration from food packaging itself. However, foods and beverages also can be contaminated prior to packaging, through handling and storage of either finished products or their ingredients. © Roy Scott

## A Silver-Bullet Lining?

Beyond reusable hard plastic bottles, the most prominent source of BPA in food-contact materials is the ubiquitous metal can. The BPA-based epoxy resin linings of cans serve a dual purpose by protecting the container from acidic or otherwise corrosive elements in foods as well as protecting food and drink from the can’s metallic taste.

Within this sector of the food-packaging industry, researchers have worked for years to identify a replacement for standard BPA-containing epoxies that performs just as well across the same range of food and beverage types.^24^ Such a coating must be physically stable and resistant to all manner of foods and beverages, and, in the case of food cans, must maintain its performance at elevated temperatures while foods are being sterilized after sealing. No replacement has yet emerged. But efforts now under way could pay dividends in the not-too-distant future.

Valspar Corporation, a Minneapolis-based manufacturer that bills itself as the number-one global supplier of coatings for metal packaging, is motivated to develop an EA-free can lining for use with a wide variety of foods. And staff toxicologist Mark Maier, who’s leading the company’s efforts, thinks he’s found it. He says Valspar has developed a replacement coating that several academic laboratories have shown to be EA-free. But even if testing validates Valspar’s invention, that doesn’t guarantee economic viability. “The supply chain challenge may be bigger than the safety challenge,” he says. “It doesn’t matter how good your technology is—if it costs too much, nobody’s going to buy it.”

Daniel Schmidt, an associate professor in the Department of Plastics Engineering at the University of Massachusetts Lowell, is leading another group in search of a new can lining. Schmidt’s lab has already made an epoxy from 2,2,4,4-tetramethyl-1,3-cyclobutanediol (CBDO), the same monomer that is at the heart of Tritan,^25^ and is working to scale it up. Funding to date has come from the university and its Toxics Use Reduction Institute, but Schmidt says a private company has recently agreed to provide support for continued research into applications that meet its needs, primarily in the beverage sector.

As to whether Schmidt’s design will ultimately show any estrogenic activity, which CertiChem’s tests on Tritan suggest it could, he admits there’s some uncertainty. “We do need to do more to ensure that everything is okay in all respects,” he says. “One of the main reasons we chose CBDO was for its structure, which bears little or no resemblance to known endocrine disruptors. This doesn’t guarantee success, but it’s a good place to start.”

Other large corporations including Dow Chemical have also alluded to their own efforts to develop safer drop-in can-lining solutions.^26^ And a number of natural and organic food brands, including Muir Glen, Eden Foods, Wild Planet, and Amy’s Kitchen, have already touted a transition to BPA-free can linings—but details are spotty as to what alternatives they’ve embraced or what level of endocrine disruption or migration the replacements represent.

Amy’s, for example, gives no information on its website as to what alternative formulation it is using, although it does say that low levels of BPA are still migrating into its food.^27^ In 1999 Eden Foods switched its linings for low-acid foods to oleoresin, a mixture of oil and resin extracted from plants such as pine and balsam fir, but high-acid foods like tomatoes are still canned with liners formulated with BPA, or bottled in jars with lids containing BPA.^28^

## Pressure up the Supply Chain

Nestlé Corporation, the world’s largest food producer with thousands of brands selling nearly any prepackaged food one can imagine, must manage the entire spectrum of food-packaging materials and their potential risks. It therefore has a considerable incentive to ensure the safety of its packaging.

The Swiss company’s food-packaging safety program got its start after a huge 2005 recall caused by the discovery that traces of isopropyl thioxanthone, a chemical used to cure packaging inks, was migrating through paper cartons into ready-to-drink baby formula sold by the company.^29^

“Nestlé got burned and said, ‘That will never happen again,’” says Stephen Klump, the company’s head of packaging quality and safety. “That was a big wake-up call for the industry.” Eventually Nestlé published guidance for inks that prohibits more than 50 acrylates, solvents, photoinitiators, and pigments.^30^ These prohibitions are based on health risks (recognized by Nestlé or perceived by the public), migration potential, and, in some cases, negative impacts on taste, smell, or color.

The company also has a policy against food contact with BPA, phthalates, and recycled paperboard, which can contain harmful chemicals derived from sources not originally intended for use in food packaging—such as newspaper ink or BPA-/BPS-containing thermal receipts that are added to recycling bins. In addition, Klump says Nestlé aims to phase out BPA from all its can linings and polycarbonate plastics by the end of 2015, but he did not specify which alternatives the company is embracing. In February of this year, Nestlé announced it is developing guidance on packaging adhesives in order to clarify its position on additional substances of concern.^31^

The company asks suppliers to formally declare compliance with its guidances as part of their contract, but does not enforce them; Klump says it can be hard to verify total compliance. Nevertheless, through these directives, Nestlé can use its sheer size to spur innovation within the food-packaging industry, and companies selling safer inks and adhesives can tout their compliance with Nestlé’s guidance as a benchmark, as SPGPrints has done with its new line of low-migration ultraviolet inkjet inks.^32^

Other large food producers hold similar sway, says Jane Muncke, managing director and chief scientific officer of the Switzerland-based Food Packaging Forum, a nonprofit foundation formed in 2012 to communicate information about food packaging and health. “They have such big buying power they’ll just switch suppliers if they’re not happy with the product.”

In this sense, the onus is often on packaging suppliers to make their products safer, which many are trying to do. A number of manufacturers have introduced new barrier films for dry foods such as pasta, cereal, and rice, among them Clondalkin Flexible Packaging,^33^ Innovia Films,^34^ Smurfit-Kappa,^35^ Imerys Kaolin,^36^ BASF,^37^ MM Karton,^38^ and Sappi Fine Paper Europe.^39^ These barriers are intended to prevent label inks and their constituent chemicals from migrating from the exterior of the package into the food, as well as stop mineral oils and other harmful substances present within recycled paper packages.^40^ Migration of mineral oils has become a significant concern for some European consumers following a European Food Safety Authority probe into the issue.^41^^,^^42^

## Incremental Changes

While it’s clear that a number of packaging manufacturers are eager to switch to alternative packaging whether required to or not, progress to date has been incremental at best. “The unqualified success may be out there, and I really do hope that these companies are developing them, but for the most part what I have seen are just-barely-studied alternatives,” says Vandenberg. Many researchers and innovators in the field who believe they’re on the right track have yet to see their eureka moment, if indeed it’s coming.

Still, change is happening. Consumer demand in Europe contributed to the development and rollout of the world’s first PVC- and plasticizer-free glass-jar lid by German packaging manufacturer Pano, says Rolf Rohrkasse, manager of product and material development for the company. Since 2011 Pano has sold 450 million of its BLUESEALÆ lids in Europe, but it has yet to break into the U.S. market. However, Pano is in discussions with Coca-Cola, Unilever, and Nestlé, among others, to expand its global reach.

The caps still contain a plastic seal—a polyolefin-based elastomer called Provalin®.^43^ While migration is not eliminated, Pano claims that migration levels are significantly lower compared with polyvinyl chloride (PVC) and its many additives.^44^ (The rubbery gaskets on almost all glass-jar lids available today contain PVC, which can leach a host of chemicals, including phthalate plasticizers, directly into foods.^45^ This is particularly true for fatty and oily foods.^46^)

However, like relying on dry-food barriers to reduce migration rather than eliminating the harmful chemicals in the first place, Muncke sees Pano’s lids as only a small step in the right direction. “It’s kind of a half-solution,” she says. “It doesn’t solve the whole issue.”

Some nongovernmental organizations are taking steps to get specific chemicals removed from food packaging.^47^ Within the last year the Natural Resources Defense Council (NRDC) has teamed with citizens’ groups in petitioning the FDA to withdraw its decades-old approvals of a handful of chemicals, including perchlorate, an endocrine disruptor used to produce rubber gaskets and to reduce static charge in plastic dry-food packaging, and long-chain perfluorocarboxylates, used to greaseproof paper and paperboard.^48^ The latter have been largely abandoned by U.S. manufacturers but increasingly are employed in India and China and are still legal to import and use, says Tom Neltner, an independent consultant.

Maricel Maffini, a consultant and former senior scientist with the NRDC, is concerned that the development of safer alternatives is being hampered by a lack of regulatory incentives and oversight. “There is no regulatory pressure for innovation,” she says. “And when [manufacturers] do take the initiative to go for an alternative, we don’t know the safety profile of that alternative, we don’t know the exposure, we don’t know if it gets metabolized when it gets into the environment. So there are still a lot of systemic improvements that we need.”

Schmidt points out that even if consumer packaging is totally free of harmful substances, there are still many opportunities during processing and handling for foods and beverages to become contaminated, even before they are packaged. As an illustration, he points to a study of phthalates in olive oil, which found contamination in every sample tested, but no significant difference in the degree of contamination between oils packaged in glass, plastic, or metal.^49^

“Packaging is important,” he says, “but the issue is even bigger still. Make the packaging perfect, and you’ve still got [contamination] coming from further up the supply chain.”

Muncke, for one, is prepared to concede that a true food-packaging panacea may not be anywhere around the next bend—especially when one takes into account the environmental impacts of producing and discarding so much packaging, and the carbon footprint of the global food system. “If you want to preserve food by using packaging, then you have to make compromises,” she says. “There is no packaging that is perfect.”

**Safety Testing**

As migration concerns drive chemists, food producers, and packaging manufactures to seek out and market new chemicals and materials, the threshold for deeming a substance “safe” is likely to become more hotly contested. Although traditional toxicity tests can be used to evaluate some outcomes of concern, endocrine disruption poses a particular challenge due to the fact that such chemicals may produce effects in experimental models at very low doses.^50^ A variety of testing regimes, tools, and assays exist to detect endocrine disruption in individual chemicals and final products. But not all are created equal, and the choice of one over another can be a matter of real consequence.

Some of the field’s leading figures in the United States, including Pete Myers of Environmental Health Sciences, Terry Collins of the Institute for Green Science at Carnegie Mellon University, and Jerrold Heindel and Thaddeus Shug of the National Institute of Environmental Health Sciences, have developed an endocrine-disruption detection system known as TiPED that is designed to help chemists formulate safer chemicals.^51^ TiPED involves a series of tests with ascending sensitivities: computational assessments, high-throughput cellular assays, cell process assays, live-animal testing with fish and amphibians, and, ultimately, mammalian testing.

Meanwhile, a European program known as LIFE-EDESIA—designed to identify three to five EA-free alternatives each for bisphenols, phthalates, and parabens—has developed a simpler *in silico* and *in vitro* tiered structure that foregoes any animal testing.^52^ And Nestlé has promoted its own computational screening method, while Valspar employs four or five assays in a tiered system that staff toxicologist Mark Maier says is essentially the same as TiPED, except it stops shy of animal testing. “The trick is when do you stop [searching for effects],” Maier says. “It just depends on who’s talking.”
