# Erratum: “A Hard Nut to Crack: Reducing Chemical Migration in Food-Contact Materials“

**DOI:** 10.1289/ehp.123-A203

**Published:** 2015-08-01

Seltenrich N. 2015. A hard nut to crack: reducing chemical migration in food-contact materials. Environ Health Perspect 123(7):A174–A179; http://dx.doi.org/10.1289/ehp.123-A174

The following corrections have been made to the figure “Packaging Pathways”: In item 5, the word “polypropylene” was changed to “polyethylene,” and in item 6, the term “non-intentionally added substances” was changed to “stabilizers.” Although it is true that the inner polyethylene layer of liquid paperboard may leach non-intentionally added substances, this is also true of all the other food-contact materials discussed in the article and should not have been implied to be unique to polyethylene.

*EHP* regrets the errors.

